# Gene Overlapping as a Modulator of *Begomovirus* Evolution

**DOI:** 10.3390/microorganisms10020366

**Published:** 2022-02-04

**Authors:** Iván Martín-Hernández, Israel Pagán

**Affiliations:** 1 Centro de Biotecnología y Genómica de Plantas UPM-INIA, 28223 Madrid, Spain; imartin@iqfr.csic.es; 2 Departamento de Biotecnología—Biología Vegetal, Escuela Técnica Superior de Ingeniería Agronómica, Alimentaria y de Biosistemas, Universidad Politécnica de Madrid, 28045 Madrid, Spain

**Keywords:** overlapping genes, rate of evolution, begomoviruses, ssDNA viruses

## Abstract

In RNA viruses, which have high mutation—and fast evolutionary— rates, gene overlapping (i.e., genomic regions that encode more than one protein) is a major factor controlling mutational load and therefore the virus evolvability. Although DNA viruses use host high-fidelity polymerases for their replication, and therefore should have lower mutation rates, it has been shown that some of them have evolutionary rates comparable to those of RNA viruses. Notably, these viruses have large proportions of their genes with at least one overlapping instance. Hence, gene overlapping could be a modulator of virus evolution beyond the RNA world. To test this hypothesis, we use the genus *Begomovirus* of plant viruses as a model. Through comparative genomic approaches, we show that terminal gene overlapping decreases the rate of virus evolution, which is associated with lower frequency of both synonymous and nonsynonymous mutations. In contrast, terminal overlapping has little effect on the pace of virus evolution. Overall, our analyses support a role for gene overlapping in the evolution of begomoviruses and provide novel information on the factors that shape their genetic diversity.

## 1. Introduction

Genomic regions that encode more than one protein, that is, gene overlapping, are commonplace among viruses [1,2]. Such regions have important biological and evolutionary implications. First, they are associated with virus within-host multiplication, between-host transmission, disease severity and strength of host immune response [3,4,5,6]. Second, viruses are subjected to strong selection for maintaining smaller genomes because this (i) reduces the chances for deleterious mutations to become fixed in the virus genome, particularly in viruses with high mutation rates; (ii) improves virus fitness due to faster replication; and (iii) optimizes virion formation due to physical limitations imposed by the capsid size [7,8,9]. Gene overlapping allows increasing the amount of genomic information in viral genomes while controlling for limited capsid space and speeding up the purification of deleterious mutations from the virus population by amplifying their effect, as in overlapping regions these mutations affect more than one gene at the same time [1,9,10].

If gene overlapping is selectively advantageous for viruses, it would be expected to be more frequent: in RNA than in DNA viruses, as the former have (in general) higher mutation rates [11]; in larger than in shorter viral genomes to minimize the chances of deleterious mutations to become fixed [12], and in spherical virions as these generally have smaller inner volumes than other capsid shapes [13]. Although Brades and Linial [9] failed to detect an association between virion shape and frequency of gene overlapping in support of the predictions above, it has been shown that the larger the gene overlapping the greater the reduction in the rate of RNA virus evolution [1], and that gene overlapping appears to be more frequent in DNA viruses, which on average have also larger genome sizes [11], than in RNA viruses [2].

**Figure 1:**
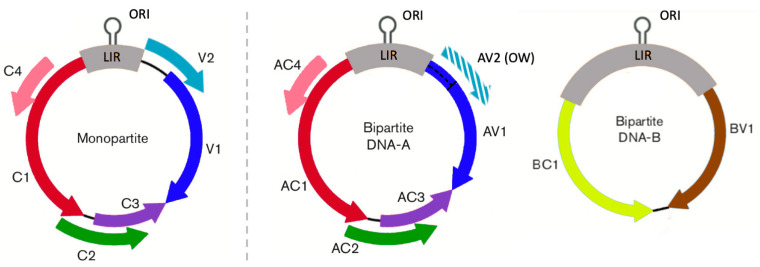
Genome organization of mono- and bipartite begomoviruses. Colored arrows denote the position and orientation of each gene. Monopartite and DNA-A of bipartite begomoviruses encode for: AC1: Replication initiator protein (Rep); AC2: Transcriptional activator protein (TrAP); AC3: Replication enhancer protein (Ren); AC4: Silencing suppressor; AV1: Coat protein (CP); and AV2: Various functions. In bipartite begomoviruses AV2 is only present in Old World (OW) begomoviruses, where AV1 is as long as in monopartite begomoviruses (dashed). DNA-B of bipartite begomoviruses encodes for: BC1: Movement protein (MP) and BC2: Nuclear shuttle protein (NSP). CR, common region. The hairpin which includes the origin of replication (ORI) is indicated in the Long Intergenic Region (LIR) (modified from [17]).

The species of the family *Geminiviridae* of plant viruses are notable exceptions to the virus characteristics associated with gene overlapping. Although geminiviruses are ssDNA viruses, and therefore replicate through high fidelity polymerases [14], and have small genomes, these viruses have a large proportion of their genes with at least one overlapping region [2]. The *Geminiviridae* family is currently divided into nine genera of which the largest one is the genus *Begomovirus*. This is also one of the most numerous genera of plant viruses with more than 400 species [15]. Bipartite begomovirus genomes encode six open reading frames (ORFs): two in the virion strand (AV1 and AV2) and four in the complementary strand (AC1, AC2, AC3 and AC4), with monopartite begomoviruses encoding equivalent proteins with the same names but without the A prefix. The AV1 gene encodes the coat protein (CP), which is essential for genome encapsidation, viral movement and insect transmission. The AV2 gene, which is considered as the pathogenicity gene, is also involved in movement and symptom development, and functions as a suppressor of gene silencing. This ORF is not present in New World bipartite begomoviruses. Viral DNA replication depends on the AC1 gene product (replication initiator protein, Rep). The AC2 gene encodes the transcriptional activator protein (TrAP) that interferes with transcriptional and post-transcriptional gene silencing (TGS and PTGS, respectively), and with the CP expression. The gene encoding for the AC3 protein (replication enhancer protein, REn), enhances viral DNA accumulation, and is involved in interaction with the plant-host retinoblastoma-related (RBR) proteins. Finally, AC4 counteracts PTGS by inhibiting accumulation of siRNA and is considered an important symptom determinant [15]. All of these six ORFs have at least one overlapping region in both mono- and bipartite begomoviruses [15]. Bipartite begomoviruses have two additional non-overlapping ORFs in the B-component: BC1, a movement protein (MP), and BV1, the nuclear shuttle protein (NSP) (Figure 1) [16].

Despite being DNA viruses, begomoviruses have been repeatedly shown to have high evolutionary rates (reviewed by [18]). For instance, *Tomato yellow leaf curl virus* (TYLCV) substitution rate has been estimated to be of 2.88 × 10^−4^ nucleotide substitutions per site per year, which is in the range of values for RNA viruses [12]. This fast evolutionary rate has been attributed to the effect of oxidative damage in replicated viral genomes, and/or to higher mutation rates than expected for DNA viruses [12]. If so, extensive gene overlapping in begomoviruses may contribute to modulate mutational load, and consequently the rate of virus evolution, as it has been shown for RNA viruses [1]. Experimental evidence supporting this idea is scarce and sometimes contradictory. For instance, a higher variability occurred in the *Tomato yellow leaf curl China virus* (TYLCCNV) AC1-AC4 overlapping (OV) region than in the non-overlapping (NOV) region of AC1 [19], whereas the opposite was observed for *Pepper huasteco yellow vein virus* (PHYVV) [20].

Here, we analyzed the effect of gene overlapping on the rate of begomovirus evolution through comparative genomics and utilizing sequences from 18 species. In particular, we explored whether the following evolutionary parameters vary between OV and NOV regions and among different types of gene overlap: (1) the rate of viral evolution, using overall tree length as a proxy, (2) the frequency of synonymous and nonsynonymous substitutions, (3) selection pressure and (4) magnitude of the effect of gene overlapping in the rate of virus evolution.

## 2. Materials and Methods

### 2.1. Sequence Data

Available sequences from begomovirus species were retrieved from GenBank. Sequences from extensively passaged isolates in non-natural hosts were excluded. When possible, we tried to minimize the presence of recombination. Species with more than 10 sequences were retained for analysis, so that we were able to include 18 mono and bipartite begomoviruses, and a total of 8239 sequences. Overall, we analyzed 125 instances of gene overlap ranging between 59 and 423 nt in length: 17 internal overlapping instances, 54 5′-terminal overlapping instances, and 54 3′-terminal overlapping instances. For simplicity, genes are named as for bipartite begomoviruses. Note that we divided sequences from *Bhendi yellow vein mosaic virus* (BYVMV) into two groups: one of sequences originally classified as belonging to this virus, and another originally characterized as *Bhendi yellow vein India virus*. Although both groups are currently considered as belonging to BYVMV [15], we chose to analyze them separately as evolutionary parameters differed between groups (Appendix A). However, analyses merging the two groups did not change our conclusions. We constructed sequence alignments for the 125 overlapping instances, and for the corresponding OV and NOV fragments of each gene. Sequence alignments of the OV regions were adjusted according to the amino acid sequence of each of the two genes involved, thus generating two data sets for each OV region. All alignments were built using MUSCLE 3.7 [21] and adjusted manually according to the amino acid sequences using AliView [22]. Alignments are available as Supplementary Material File S1.

### 2.2. Estimation of Tree Length

Tree lengths (*t*) were estimated for the OV and NOV regions of each gene. To do so, we used a maximum likelihood fitting of the General Time Reversible (GTR) nucleotide substitution model as implemented in the HyPhy package [23]. Differences in total tree length between OV and NOV regions were analyzed using a relative ratio test also utilizing HyPhy. Because *t* is dependent on the number of tree branches (i.e., number of sequences), when values were compared among overlapping instances, *t* was normalized according to the number of sequences.

### 2.3. Selection Pressures

Selection pressures for OV and NOV regions were estimated as the difference between the mean number of nonsynonymous (*d_N_*) and synonymous (*d_S_*) nucleotide substitutions per site (*d_N_*/*d_S_*) using the fast unbiased Bayesian approximation (FUBAR), and the fixed effect likelihood (FEL) methods implemented in HyPhy (Appendix A). Because the two methods yielded similar results, only the FUBAR results are shown here. In all cases, *d_N_*/*d_S_* measures were based on neighbor-joining trees inferred using the MG94 nucleotide substitution model. Significant differences between *d_N_*/*d_S_* values in OV and NOV regions, were analyzed using a population level adaptation test [24]. Values of *d_N_* and *d_S_* were also estimated. For each pair of overlapping genes, *d_N_*, *d_S_*, and *d_N_*/*d_S_* estimates were obtained for the two reading frames of the OV region. To do so, we used separated sequence alignments for the two overlapping genes, and we partitioned codons, such that OV and NOV regions could be defined over the full-length sequence of each gene.

### 2.4. Detection of Recombination

For each pair of overlapping genes, recombination breakpoints were detected using six different methods as implemented in RDP5: RDP, GENECONV, MaxChi, 3Seq, Bootscan, and Chimaera [25]. Only recombination signals detected by at least four methods (*p* < 0.05) were considered as positive. For the purpose of this work, recombinants with breakpoints in the LIR and the V1/C3 limit, which are recombination hotspots [26], were not counted as such as they were not differentially affecting OV and NOV regions of any given gene. Instances with more than 10% of recombinant sequences, regardless of breakpoints were located in OV or NOV regions, were considered to have excessive recombination (Appendix A). Analyses were repeated excluding such instances, but conclusions did not vary. Hence, we present here results obtained using all instances.

### 2.5. Statistical Analysis

The 125 overlapping instances were used for statistical analysis. Tree lengths (*t*) were not homoscedastic according to Kolmogorov–Smirnov and Levene’s tests. Therefore, this variable was fitted to a gamma distribution; whereas the ratio OV/NOV for *t*, *d_N_*, *d_S_*, and *d_N_*/*d_S_*, and percentage of overlapping were fitted to a normal distribution, according to Akaike’s Information Criteria (R package: RRISKDISTRIBUTIONS; [27]). Consequently, differences in values of these variables between OV and NOV regions and between types of gene overlap were analyzed by generalized linear models (GzLM), using type of region or type of overlapping as factors. Differences in the proportion of genes for which parameters above differed between OV and NOV regions was analyzed by Fisher’s exact test [28]. Associations between parameters were tested using Pearson´s correlation tests. All statistical analyses were performed using the statistical software packages SPSS 17.0 (SPSS Inc., Chicago, IL, USA) and R v.3.6.3 [29].

## 3. Results

### 3.1. Effect of the Presence and Type of Gene Overlapping on Gene Evolution

**Figure 2:**
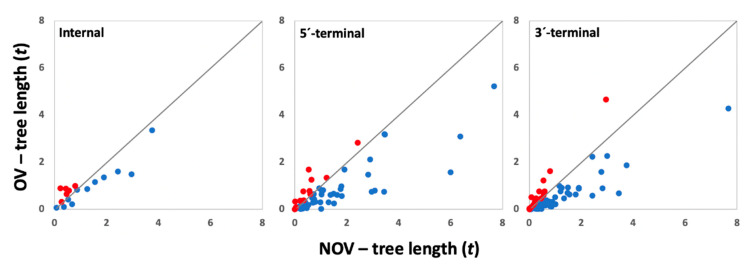
Overlapping and nonoverlapping tree lengths (*t*) in overlapping genes. Blue dots denote genes in which *t* is significantly higher in NOV than in OV regions. Red dots denote genes showing the opposite trend. Genes with different types of overlapping (internal, 5′-, and 3′-terminal) are presented in different panels.

We analyzed the effect of gene overlapping on the rate of begomovirus evolution by estimating the total length of the tree (*t*) inferred for the OV and NOV regions of each gene (Figure 2). In OV regions, *t* ranged from 0.001 to 5.218, depending on the gene–virus combination, with mean value of 0.797 (median: 0.573). Variation in *t* for NOV regions ranged between 0.006 and 7.676, with mean value of 1.262 (median: 0.744). A GzLM analysis using type of region (OV and NOV) as a factor indicated that *t* was significantly smaller in OV than in NOV regions (Wald *χ^2^* = 10.74; *p* = 1 × 10^−3^). In agreement with these results, in most overlapping instances, *t* was significantly smaller in OV than in NOV regions (93/125, *χ^2^* = 59.54, *p* < 1 × 10^−5^) (Figure 2 and Appendix A). Hence, gene overlapping generally reduces evolutionary rates. However, viruses can generate different types of gene overlap, which arise by different mechanisms and that generally differ in the resulting frameshift [10] and the degree of selective independence of the genes involved [7]. Therefore, it could be hypothesized that evolutionary rates differ by type of gene overlap.

Thus, three types of gene overlap were defined following [1]: (1) internal overlapping, when one of the genes contains the complete sequence of the other; (2) 5′-terminal overlapping, when the OV region is in the 5′-terminal region of the gene; and (3) 3′-terminal overlapping, when the OV region is in the 3′-terminal region of the gene. Genes with terminal overlapping showed significantly lower *t* values in OV than in NOV regions (Wald *χ^2^* ≥ 4.88; *p* ≤ 0.027), with most instances fitting this general observation (42/54, *χ^2^* = 33.33, *p* < 1 × 10^−5^; and 39/54, *χ^2^* = 21.33, *p* < 1 × 10^−5^, for 5′- and 3′-terminal overlapping, respectively). In contrast, in genes with internal overlapping no significant differences between OV and NOV regions were observed (Wald *χ^2^* ≤ 1.99; *p* ≥ 0.212), and instances with lower *t* in OV regions were not significantly more frequent (11/17, *χ^2^* = 2.94, *p* = 0.086) (Figure 2 and Appendix A). We also analyzed differences in the magnitude of the effect of each type of overlapping in reducing the rate of virus evolution. For that, we calculated the OV/NOV ratio for *t* values of overlapping instances where this parameter was significantly smaller in OV regions. A GzLM indicated that the magnitude of the effect on *t* depended on the type of overlapping (Wald *χ^2^* = 3.71; *p* = 0.028), with terminal ones showing similar effects (*p* = 0.314) and in both cases higher than internal overlapping (*p* ≤ 0.041). Same conclusions were obtained when normalized *t* values were used.

Our dataset included mono- and bipartite begomoviruses, which differ in host-virus and virus-virus protein-protein interactions [30]. This may result in differential evolutionary constraints that may modulate how gene overlapping affects virus evolution. Thus, we analyzed whether gene overlapping influenced tree length depending on the begomovirus genome structure. GzLMs using this trait (mono- vs. bipartite) as a factor indicated that it had no effect on *t* differences between OV and NOV regions (Wald *χ^2^* = 2.24; *p* = 0.137). In agreement, *t* was significantly higher in NOV than in OV regions when mono- and bipartite begomoviruses were analyzed separately (Wald *χ^2^* = 4.30; *p* = 0.038 and Wald *χ^2^* = 8.67; *p* = 3 × 10^−3^, respectively). In both groups of viruses, the same was observed when each type of terminal overlapping was analyzed separately (Wald *χ^2^* ≥ 4.90; *p* ≤ 0.027), but not for internal overlapping (Wald *χ^2^* ≤ 0.82; *p* ≥ 0.366). The proportion of instances with higher *t* in NOV that in OV regions was higher than expected by chance in terminal overlapping of both types of genome structures (*χ^2^* ≥ 4.92, *p* ≤ 0.026), but not in internal overlapping (*χ^2^* ≤ 0.98, *p* ≥ 0.173) (Appendix A).

In sum, these results indicate that the effect of gene overlapping on the rate of begomovirus evolution varies depending on its type; terminal overlapping generally reduces tree length, whereas no clear trend is observed in genes with internal overlapping. On the other hand, the type of genomic structure has little effect on the observed patterns.

### 3.2. Association between Selection Pressures and Gene Evolution

**Figure 3:**
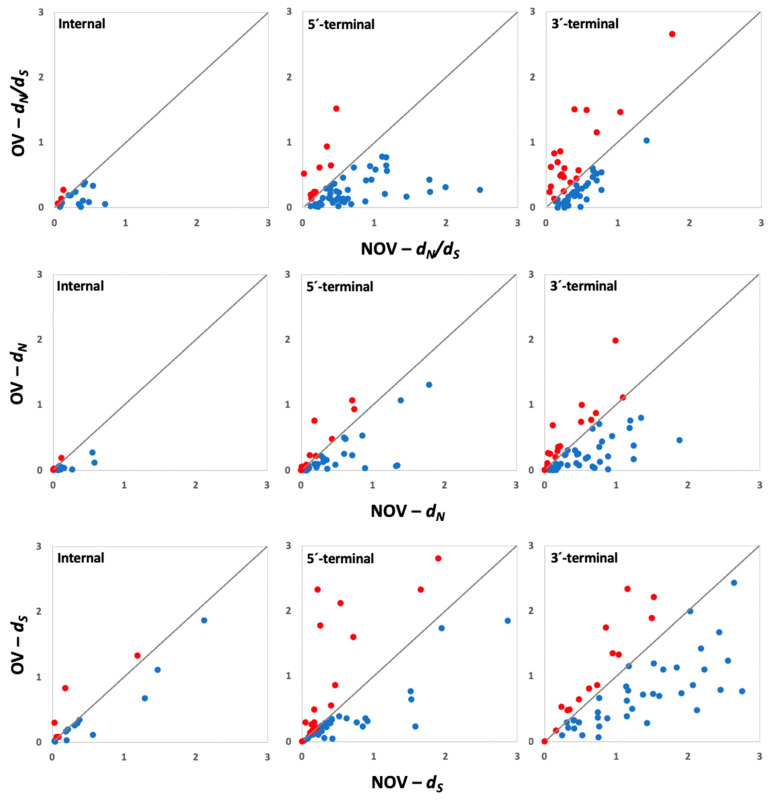
Overlapping and nonoverlapping *d_N_/d_S_* (upper line), *d_N_* (middle line) and *d_S_* (lower line) in overlapping genes. Blue dots denote instances in which parameters are significantly higher in NOV than in OV regions. Red dots denote genes showing the opposite trend. Genes with different types of overlapping (internal, 5′-, and 3′-terminal) are presented in different panels.

To further analyze how gene overlapping reduced the rate of evolution, we estimated selection pressures (*d_N_/d_S_*) and individual *d_N_* and *d_S_* values for the OV and NOV regions of each gene (Figure 3 and Appendix A). Average *d_N_/d_S_* values were 0.35 ± 0.03 and 0.50 ± 0.04 for OV and NOV regions, respectively. A GzLM using type of region as factor indicated that negative selection pressures were significantly stronger in OV than in NOV regions (Wald *χ^2^* = 11.42; *p* < 1 × 10^−5^), and we obtained similar results when each type of overlap was analyzed independently (Wald *χ^2^* ≥ 8.18; *p* ≤ 2 x10^−4^, Figure 3 and Appendix A). In agreement, most genes had significantly higher *d_N_/d_S_* in NOV than in OV fragments when all types of overlap were considered together (89/125, *χ^2^* = 44.94, *p* < 1 × 10^−5^) and for the three of them independently (14/17, *χ^2^* = 14.24, *p* = 1.6 × 10^−4^; 43/54, *χ^2^* = 37.93, *p* < 1 × 10^−5^; 33/54, *χ^2^* = 5.33, *p* = 0.021, for internal, 5′-, and 3′-overlapping, respectively) (Figure 3 and Appendix A).

Similar analysis for *d_N_* indicated significantly lower values in OV than in NOV regions when all genes were considered together (0.24 ± 0.03 and 0.39 ± 0.04, respectively; Wald *χ^2^* = 12.10; *p* < 1 × 10^−5^), and when each type of overlapping was analyzed separately (Wald *χ^2^* ≥ 9.21; *p* ≤ 5 × 10^−3^). Also, in most instances, *d_N_* followed this trend (92/125, *χ^2^* = 55.70, *p* < 1 × 10^−5^), with similar results for each type of overlap (13/17, *χ^2^* = 7.53, *p* = 6.1 × 10^−3^; 42/54, *χ^2^* = 33.33, *p* < 1 × 10^−5^; 37/54, *χ^2^* = 14.81, *p* = 1.2 × 10^−4^, for internal, 5′-, and 3′-overlapping, respectively) (Figure 3). Finally, *d_S_* was similar in NOV and in OV regions either considering all genes together (0.72 ± 0.08 and 0.83 ± 0.07, respectively; Wald *χ^2^* = 2.16; *p* = 0.079) or analyzing each type of overlap independently (Wald *χ^2^* ≤ 3.18; *p* ≥ 0.101) (Figure 3). However, instances with *d_S_* value higher in NOV than in OV regions were more frequent than expected by chance (86/125, *χ^2^* = 35.34, *p* < 1 × 10^−5^), with similar results for each type of overlap (12/17, *χ^2^* = 5.76, *p* = 0.016; 35/54, *χ^2^* = 9.48, *p* = 2.1 × 10^−3^; 39/54, *χ^2^* = 21.33, *p* < 1 × 10^−5^, for internal, 5′-, and 3′-overlapping, respectively) (Figure 3).

When mono- and bipartite begomoviruses were analyzed separately, *d_N_/d_S_* and *d_N_* (Wald *χ^2^* ≥ 7.70; *p* ≤ 6 × 10^−3^, Wald *χ^2^* ≥ 4.18; *p* ≤ 0.041, respectively), but not *d_S_* (Wald *χ^2^*≤ 3.00; *p* ≥ 0.083), were always higher in NOV than in OV regions for viruses with both genomic structures. When each type of overlapping was analyzed separately, similar results were obtained for *d_N_/d_S_* and *d_N_* (Wald *χ^2^* ≥ 4.11; *p* ≤ 0.043, Wald *χ^2^* ≥ 6.61; *p* ≤ 0.010 and Wald *χ^2^* ≥ 3.60; *p* ≤ 0.050, for internal, 5′-, and 3′-overlapping, respectively), and for *d_S_* (Wald *χ^2^* ≥ 0.20; *p* ≤ 0.652, Wald *χ^2^* ≥ 1.20; *p* ≤ 0.274 and Wald *χ^2^* ≥ 2.66; *p* ≤ 0.103, for internal, 5′-, and 3′-overlapping, respectively). As above, the proportion of overlapping instances with higher *d_N_/d_S_*, *d_N_* and *d_S_* in NOV than in OV regions was generally larger than those showing the opposite trend in viruses with both types of genome structure and in all types of gene overlapping (*χ^2^* ≥ 3.63, *p* ≤ 0.050) (Appendix A).

Thus, overlapping genes are generally subjected to stronger purifying selection in OV than in NOV fragments, which seems to be associated with a greater constraint against non-synonymous changes regardless of the type of overlap and, to a lesser extent, with constraints to synonymous changes. Again, the type of genomic structure had no influence in the observed results.

### 3.3. Association between Proportion of Overlap and Gene Evolution

**Figure 4:**
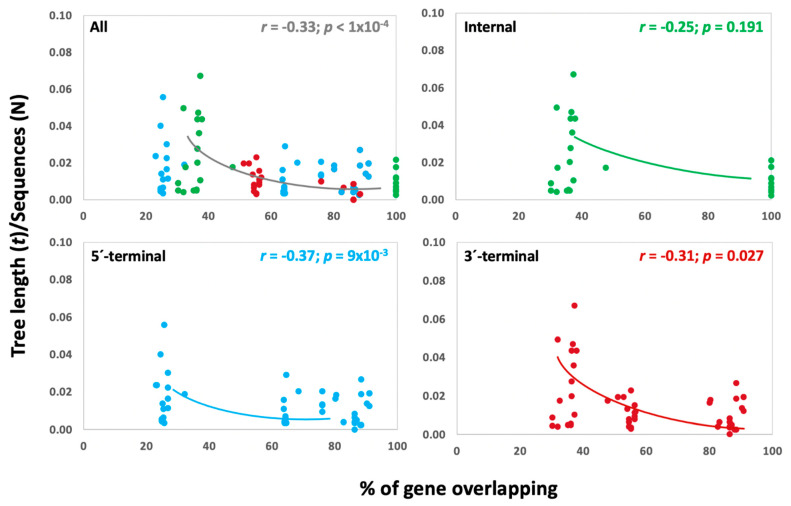
Correlation between tree length (*t*/number of sequences) and the proportion of gene overlap (length of overlap/total gene length) for all types of overlap considered together (**upper left**), internal overlapping (**upper right**), 5′-terminal overlapping (**lower left**) and 3′-terminal overlapping (**lower right**).

For RNA viruses, it has been shown that the lengths of the OV region relative to gene length are negatively correlated with these rates in a non-linear manner [1,31]. We analyzed whether this relationship held for begomoviruses by calculating the normalized tree length for the complete sequence of each gene and assessing the strength of association between *t* and the proportion of gene overlap (Figure 4). As the genome structure had no effect in previous analyses, we did not consider this trait here. On the other hand, we included normalized tree lengths for AC4 (100% overlap), which were not considered previously as in this gene no OV vs. NOV comparison was possible.

The proportion of gene overlap (%) differed among types of overlap (Wald *χ^2^* = 8.74; *p* < 1 × 10^−4^): it was lower in genes with internal (35.67 ± 0.97) than terminal overlapping (60.59 ± 3.65 and 59.90 ± 3.18 for 5′- and 3′-terminal overlapping, respectively). Hence, we analyzed the association between per cent of gene overlap and *t* in the complete sequence of each gene for all genes together and for each type of overlap separately. We performed bivariate analysis considering linear and nonlinear regressions. When a significant association was found, it was best explained by a negative logarithmic relationship between the length of overlap and *t* (Figure 4). Bivariate analysis revealed a significant negative logarithmic association between these two variables when all instances were considered together (*r* = −0.33; *p* < 1 × 10^−4^; Figure 4), with similar results when excluding values for AC4 (*r* = −0.32; *p* < 1 × 10^−4^). We also found a significant negative logarithmic association in both types of terminal overlap (*r* = −0.37, *p* = 9 × 10^−3^ and *r* = −0.31, *p* = 0.027; for 5′-, and 3′-overlapping, respectively), but not for internal ones with (*r* = −0.25, *p* = 0.191; Figure 4) and without (*r* = 0.23, *p* = 0.383) AC4 values. Comparable results were obtained using only those genes for which *t* values were higher in NOV than in OV regions.

## 4. Discussion

Several non-mutually exclusive theories have been proposed to explain the abundance of gene overlapping in viruses: (i) it has a role in gene regulation by providing an inherent mechanism for coordinated expression [7]; (ii) it is an effective mechanism for generating novel genes while keeping genome size minimized, by introducing a new reading frame on top of an existing one [32,33]; or (iii) as mutations in these regions affect more than one gene, gene overlapping amplifies the deleterious effect of mutations, thus quickly eliminating such mutations from the viral population, particularly in RNA viruses which have higher mutation rates [7,34,35]. Although there is general agreement on the role of gene overlapping in maintaining genomic compression [10,31,36,37], its effect on virus evolutionary rates remains more elusive [1,2]. This is particularly so for DNA viruses that despite having in general lower mutation rates than RNA viruses have in some cases larger proportion of their genes with at least one overlapping instance [2]. Here, we analyzed whether in the largest genus of plant DNA viruses, whose genome is enriched in gene overlapping instances, this feature modulates the rate of gene evolution.

Our comparative genomic analyses in species of the genus *Begomovirus* indicate that tree length (as a proxy of the rate of evolution) was generally smaller in OV than in NOV regions, with most overlapping instances following this rule. This agrees with the predictions of mathematical models [34,38,39]. Interestingly, these models also predict that the reduction in evolutionary rate is the consequence of correlations at overlapping sites, which are stronger in positions where a mutation would result in a nonsynonymous change in both overlapping genes than in positions where mutations are synonymous in one gene and nonsynonymous in the other [7,34,38]. This may explain why our results indicate that the reduction in the genetic diversity of OV regions is associated with decreased *d_N_*, but not *d_S_* although in most instances OV regions had lower values of both parameters: gene overlapping would influence both synonymous and nonsynonymous substitution, but this effect would be stronger in nonsynonymous ones. There was significant negative (logarithmic) correlation between the length of overlap and the genetic diversity of each gene; that is, the longer the OV region, the lower the evolutionary rate. This agrees with theoretical models, which predict that evolutionary rate is expected to decline nonlinearly with increasing overlap [7]. This negative logarithmic association also indicates that an increased proportion of gene overlapping reduces begomovirus evolutionary rates up to a threshold, beyond which larger overlapping has no effect on tree length. Thus, long overlapping regions cannot be fully explained by their effect on evolutionary rates alone, and other selection pressures, such as genome compression or coordinated gene expression are likely to play a role.

Altogether, our results provide compelling evidence supporting the role of gene overlapping in reducing the rate of *Begomovirus* evolution. This observation is in accordance with previous reports for a variety of RNA viruses [1,40,41,42]. In most of these cases, the reduction of the rate of virus evolution associated with gene overlapping has been attributed to the need of these viruses to buffer excessive mutational load due to high mutation rates. To date, however, estimates of mutation rates in DNA viruses suggest that these are lower than for RNA viruses [11]. Two lines of evidence suggest that this might not be the case for begomoviruses. First, rough estimates of mutation frequency in TYLCCNV showed values around 1 × 10^−4^ [19], which is comparable to the variation reported for plant RNA viruses and higher than for other ssDNA viruses [11,43]. Second, it has been shown that some of the DNA polymerases involved in begomovirus replication are error-prone in conditions equivalent to those in which they amplify the viral genome [44,45]. Hence, begomoviruses could have evolved overlapping regions as a safety mechanism to control high mutation rates.

Evolutionary constraints imposed by gene overlapping are a double-edged sword. They restrict the fixation of deleterious mutations; but at the same time, they leave little room to increase virus fitness, as beneficial mutations in one gene are often deleterious in the other and are therefore purged [1,4]. Viruses are faced with the need to reconcile these two facets such that they limit the fixation of unfit mutations but allow generation of beneficial genetic diversity. To do so, it has been shown that viruses may use a “segregated” organization in which overlapped regions harbor functional domains of one gene or the other, but never both [4]. Thus, gene overlapping imposes a certain degree of evolutionary constraint, as mutations affect more than one gene at the same time. However, this is not as strong as if both genes would harbor functional domains in the overlapping region, or as relaxed as if both genes would not overlap. This strategy results in higher fitness peaks than in the absence of gene overlapping [4]. Interestingly, some evidence suggests that begomoviruses may use a similar strategy. For instance, AV1 functional domains involved in DNA shuttle into the nucleus or in vector transmission are located at the N-terminal region of the protein, overlapping with AV2 [46]; whereas hydrophobic domains involved in the silencing suppression activity of AV2 locate at the NOV region of this protein [47]. Similarly, in AC2 the domain responsible for repressing AV1 expression is in the NOV region of this gene [48], whereas the OV region of AC3 is rich in functional domains [47].

Despite the general trend toward a reduction of genetic diversity in OV compared with NOV regions, when each type of overlapping was analyzed separately, this effect remained significant only in instances with 5′- and 3′-terminal overlap, whereas nearly one-third of the instances with internal overlap showed the opposite trend. Different types of overlapping vary in the preponderance of the associated frameshifting [10]. However, in begomoviruses all overlapping instances have +1/−1 frameshift, which are identical in the extent to which they allow selective independence of the overlapping genes [7]. Alternatively, in our dataset we included mono- and bipartite begomoviruses, for which different functions have been attributed to the C4/AC4 proteins [30]. Different selective pressures on C4/AC4 depending on its function may impose different constraints on its evolution, modulating the buffering effects of gene overlapping on the accumulation of mutations on AC1. We do not favor this hypothesis as our results indicate that genomic structure has little effect on the role of gene overlapping as modulator of begomovirus evolution. Another possible explanation for the observed differences is that, as we restricted our analyses to a single virus genus and each type of overlap occurs in the same genes across species, differences between terminal and internal overlapping reflect particular characteristics of the genes involved. Indeed, internal overlapping instances involved the same two genes (AC1 and AC4) in all species. If, for instance, the AC1 gene is dominating the evolution of AC4, as has been shown for younger overlapping genes generated by overprinting over older ones [33,49], the resulting internal overlapping would have less effect in the evolution of AC1, in accordance with our results. In addition, note that AC1 is involved in virus replication, which is a key component of virus fitness, thus this gene is more likely to drive AC4 evolution rather than the other way around. In support of this hypothesis, it has been shown that AC1 is under strong negative selection, whereas AC4 is under positive selection [50]. Finally, at odds with the examples mentioned above, functional conserved domains are not segregated in AC1/AC4 [47,51], which would also support that the observed differences respond to gene-specific features.

An additional source of gene-specific heterogeneity in our dataset that could explain the differential effect of internal and terminal overlapping in begomovirus evolution is the presence of recombination. Large fragments of AC1 (including the region overlapping with AC4) are recombination hotspots, whereas AC2/AC3 and big portions of AV1 and AV2 are coldspots [26,52]. It has been hypothesized that recombination allows removing deleterious mutations with high efficiency, as reviewed by [53]. Hence, the limited effect of AC1/AC4 internal overlapping in virus evolutionary rates could be explained by a higher frequency of recombination in AC1, which in NOV regions would have similar consequences than gene overlapping. Although we cannot completely discard such a role of recombination, at least in our dataset several observations argue against it. First, the percentage of instances with over 10% of recombinant sequences was evenly distributed across types of overlapping (31–35%, Appendix A). If recombination in AC1/AC4 were to explain our results, we would have expected more frequent recombination in internal than in terminal overlapping. Rather, virus species identity seemed to explain most of the variation in recombination frequency, with three species (*Bhendi yellow vein mosaic virus*, *Chilli leaf curl virus* and *Okra enation leaf curl virus*) accounting for two thirds of the overlapping instances with excessive recombination. Second, when these instances (41/125) were removed from the analyses, we still observed higher *t* values in NOV than in OV regions in terminal (30/37 and 27/36 instances, for 5′-, and 3′-terminal overlapping, respectively), but not in internal (5/10 instances), overlapping. Hence, our conclusions hold regardless of the presence of extensive recombination.

Some cautionary comments on our results are called for, however. First, the number of instances among types of overlapping is not fully balanced, with lower numbers for internal than for both types of terminal overlap. Hence, the lack of a significant effect of OV regions in internal overlap could be due to reduced sample size. Second, because we restricted our analyses to a single virus genus, overlapping instances occur in the same genes across species, which may reduce the range of overlapping lengths included in the regression analyses with the subsequent reduction of statistical power. However, the range of terminal overlapping lengths included was enough to detect a significant correlation between percent of gene overlapping and genetic diversity. This range was much smaller for internal overlapping, which again may explain the lack of association between the two analyzed traits. Finally, we could include only 18 out of the 420 begomovirus species, as these were the only ones that fulfilled the criteria to be included in our analyses. Despite the small sample size, our results indicate strongly significant effects, which support the relevant role of gene overlapping in begomovirus evolution.

In sum, this work provides novel evidence of the selective constraints imposed by gene overlapping on the pace of begomovirus evolution. Whether this effect is general for DNA viruses would be an interesting avenue of future research.

## Data Availability

Data available as Supplementary Material and as Appendix A.

## Supplementary Materials

The following are available online at https://www.mdpi.com/article/10.3390/microorganisms10020366/s1, File S1: Sequence alignments used in this work.

## Appendix A

**Table A1:** Statistical parameters of traits associated with the rate of evolutionary change in overlapping genes.

Species ^1^	N ^2^	Overlapping ^3^		Gene Length ^4^	*t* ^5^		*d_N_/d_S_*	
		ORF	Length		NOV	OV	NOV	OV
Internal (11/17)								
*African cassava mosaic virus (B)*	27 (4%)	AC1-AC4	423	1077	0.53	0.41	0.39	0.11
*Alternanthera yellow vein virus (M)*	11 (8%)	AC1-AC4	291	1086	0.47	0.64	0.54	0.33
*Bean golden mosaic virus (B)*	121 (0%)	AC1-AC4	258	1086	0.44	0.86	0.34	0.06
*Bhendi yellow vein mosaic virus* (M)*	39 (98%)	AC1-AC4	294	1092	1.25	0.85	0.48	0.09
*Bhendi yellow vein mosaic virus (M)*	32 (99%)	AC1-AC4	303	1092	1.90	1.35	0.11	0.08
*Chilli leaf curl virus (M)*	16 (28%)	AC1-AC4	300	1086	0.86	0.83	0.22	0.20
*Cotton leaf curl Gezira virus (M)*	21 (9%)	AC1-AC4	294	1089	0.22	0.89	0.37	0.02
*Cotton leaf curl Multan virus (M)*	50 (2%)	AC1-AC4	303	1092	0.67	0.21	0.07	0.02
*East African cassava mosaic Kenya virus (B)*	50 (0%)	AC1-AC4	297	1065	0.27	0.31	0.05	0.07
*East African cassava mosaic Malawi virus (B)*	13 (0%)	AC1-AC4	234	1080	0.07	0.06	0.20	0.19
*East African cassava mosaic virus (B)*	153 (2%)	AC1-AC4	234	1080	1.55	1.15	0.41	0.36
*East African cassava mosaic Zanzibar virus (B)*	14 (29%)	AC1-AC4	258	1080	0.35	0.09	0.08	0.03
* Okra enation leaf curl virus (M)*	60 (92%)	AC1-AC4	308	1089	2.96	1.48	0.29	0.24
* South African cassava mosaic virus (B)*	125 (0%)	AC1-AC4	297	1080	0.55	0.79	0.09	0.14
* Sweet potato leaf curl virus (M)*	17 (41%)	AC1-AC4	258	1095	0.79	0.99	0.12	0.28
* Tomato leaf curl New Delhi virus (B)*	97 (3%)	AC1-AC4	303	1086	3.75	3.34	0.43	0.39
* Tomato yellow leaf curl virus (M)*	397 (68%)	AC1-AC4	294	1074	2.43	1.60	0.70	0.05
5′-terminal (42/54)								
*African cassava mosaic virus (B)*	32 (3%)	AC1-AC2	93	408	0.21	0.37	0.11	0.20
	31 (3%)	AC2-AC3	260	405	0.31	0.03	0.50	0.03
	26 (0%)	AV1-AV2	193	777	0.39	0.26	0.37	0.15
*Alternanthera yellow vein virus (M)*	11 (18%)	AC1-AC2	98	406	0.22	0.01	0.22	0.02
	13 (0%)	AC2-AC3	260	405	0.71	0.27	0.11	0.03
	10 (60%)	AV1-AV2	189	771	0.33	0.76	1.15	0.21
*Bean golden mosaic virus (B)*	158 (0%)	AC1-AC2	89	390	1.30	0.29	0.40	0.13
	158 (0%)	AC2-AC3	254	399	1.50	0.25	0.26	0.05
*Bhendi yellow vein mosaic virus* (M)*	51 (71%)	AC1-AC2	104	453	1.91	1.68	0.33	0.29
	51 (20%)	AC2-AC3	308	405	3.44	0.74	0.28	0.14
	50 (40%)	AV1-AV2	206	771	1.76	0.85	0.63	0.14
*Bhendi yellow vein mosaic virus (M)*	57 (54%)	AC1-AC2	104	453	2.42	2.82	1.01	0.58
	57 (2%)	AC2-AC3	308	405	1.80	0.56	1.18	0.56
	56 (16%)	AV1-AV2	206	771	1.63	0.61	0.38	0.21
*Chilli leaf curl virus (M)*	18 (33%)	AC1-AC2	98	405	0.99	0.30	0.17	0.02
	23 (17%)	AC2-AC3	260	405	3.08	0.79	0.47	0.25
	22 (18%)	AV1-AV2	197	771	1.22	1.33	0.18	0.24
*Cotton leaf curl Gezira virus (M)*	32 (6%)	AC1-AC2	101	405	0.01	0.33	0.02	0.52
	32 (0%)	AC2-AC3	257	402	0.47	0.04	0.42	0.07
	31 (0%)	AV1-AV2	209	777	0.35	0.37	0.16	0.24
*Cotton leaf curl Multan virus (M)*	58 (2%)	AC1-AC2	104	453	0.59	0.65	0.12	0.14
	59 (2%)	AC2-AC3	308	405	1.08	0.80	1.11	0.78
	59 (15%)	AV1-AV2	206	771	1.04	0.75	0.44	0.37
*East African cassava mosaic Kenya virus (B)*	71 (0%)	AC1-AC2	77	408	0.40	0.29	0.25	0.08
	71 (0%)	AC2-AC3	260	405	0.34	0.22	0.18	0.06
	64 (0%)	AV1-AV2	197	774	0.23	0.18	0.55	0.10
*East African cassava mosaic Malawi virus (B)*	9 (0%)	AC1-AC2	92	408	0.00	0.00	-	-
	10 (0%)	AC2-AC3	260	405	0.06	0.03	0.57	0.14
	12 (0%)	AV1-AV2	191	777	0.05	0.08	0.17	0.22
*East African cassava mosaic virus (B)*	166 (2%)	AC1-AC2	92	405	1.79	0.97	2.47	0.27
	162 (0%)	AC2-AC3	260	405	1.38	0.60	0.67	0.05
	105 (0%)	AV1-AV2	197	775	0.79	0.31	0.58	0.09
*East African cassava mosaic Zanzibar virus (B)*	15 (27%)	AC1-AC2	92	408	0.23	0.01	0.50	0.15
	15 (0%)	AC2-AC3	260	405	0.11	0.10	1.76	0.42
	15 (0%)	AV1-AV2	197	775	0.20	0.07	0.63	0.27
*Okra enation leaf curl virus (M)*	67 (97%)	AC1-AC2	104	453	3.45	3.18	0.51	0.23
	68 (32%)	AC2-AC3	308	405	1.04	0.63	1.44	0.17
	41 (58%)	AV1-AV2	188	771	0.64	1.26	0.24	0.62
*Pepper golden mosaic virus (B)*	54 (57%)	AC1-AC2	59	390	1.49	0.66	0.47	0.12
	54 (0%)	AC2-AC3	254	399	0.94	0.89	0.34	0.94
*Pepper huasteco yellow vein virus (B)*	19 (5%)	AC1-AC2	80	417	1.01	0.01	0.38	0.28
	45 (0%)	AC2-AC3	254	399	0.74	0.42	1.99	0.32
*South African cassava mosaic virus (B)*	131 (0%)	AC1-AC2	92	408	0.56	0.77	0.47	1.52
	130 (0%)	AC2-AC3	260	405	0.67	0.48	0.40	0.64
	126 (4%)	AV1-AV2	191	777	0.73	0.64	1.17	0.65
*Sweet potato leaf curl virus (M)*	18 (39%)	AC1-AC2	92	452	0.54	1.68	0.39	0.33
	14 (7%)	AC2-AC3	278	435	2.96	0.73	1.78	0.25
	18 (9%)	AV1-AV2	176	765	0.52	0.17	0.87	0.10
*Tomato leaf curl New Delhi virus (B)*	88 (4%)	AC1-AC2	98	420	2.83	1.47	0.71	0.62
	113 (1%)	AC2-AC3	281	411	5.99	1.56	0.57	0.46
	115 (0%)	AV1-AV2	179	771	2.90	2.12	1.16	0.77
*Tomato yellow leaf curl virus (M)*	588 (9%)	AC1-AC2	92	408	7.68	5.22	0.96	0.43
	521 (10%)	AC2-AC3	260	405	6.37	3.08	0.95	0.64
	593 (10%)	AV1-AV2	191	777	3.47	3.16	0.89	0.42
3′-terminal (39/54)								
*African cassava mosaic virus (B)*	27 (3%)	AC1-AC2	93	1077	0.53	0.36	0.50	0.29
	32 (3%)	AC2-AC3	260	408	0.21	0.03	0.44	0.20
	33 (0%)	AV1-AV2	193	342	0.54	0.24	0.47	0.18
*Alternanthera yellow vein virus (M)*	11 (18%)	AC1-AC2	98	1086	0.47	0.01	0.48	0.02
	11 (0%)	AC2-AC3	260	406	0.22	0.27	0.45	0.57
	12 (60%)	AV1-AV2	189	348	1.21	0.76	0.57	0.13
*Bean golden mosaic virus (B)*	121 (0%)	AC1-AC2	89	1086	0.44	0.10	0.56	0.34
	158 (0%)	AC2-AC3	254	390	0.30	0.28	0.65	0.61
*Bhendi yellow vein mosaic virus* (M)*	39 (92%)	AC1-AC2	104	1092	1.25	0.91	0.58	0.38
	51 (20%)	AC2-AC3	308	453	1.91	0.91	0.27	0.03
	50 (40%)	AV1-AV2	206	366	1.34	0.46	0.13	0.10
*Bhendi yellow vein mosaic virus (M)*	23 (98%)	AC1-AC2	104	1092	1.90	0.88	0.16	0.70
	57 (2%)	AC2-AC3	308	453	2.42	0.57	0.21	0.51
	55 (16%)	AV1-AV2	206	366	0.89	0.32	0.78	0.54
*Chilli leaf curl virus (M)*	16 (38%)	AC1-AC2	98	1086	0.86	0.10	0.30	0.12
	18 (17%)	AC2-AC3	260	405	0.99	0.50	0.66	0.57
	16 (19%)	AV1-AV2	197	357	0.26	0.45	1.76	2.67
*Cotton leaf curl Gezira virus (M)*	21 (9%)	AC1-AC2	101	1089	0.22	0.33	0.07	0.33
	32 (0%)	AC2-AC3	257	405	0.01	0.03	0.24	0.47
	29 (0%)	AV1-AV2	209	369	0.08	0.50	0.07	0.63
*Cotton leaf curl Multan virus (M)*	50 (2%)	AC1-AC2	104	1092	0.67	0.38	0.27	0.03
	58 (2%)	AC2-AC3	308	453	0.59	0.75	0.11	0.14
	57 (16%)	AV1-AV2	206	366	0.45	0.46	0.24	0.26
*East African cassava mosaic Kenya virus (B)*	50 (0%)	AC1-AC2	77	1065	0.27	0.01	0.38	0.25
	71 (0%)	AC2-AC3	260	408	0.40	0.25	0.25	0.61
	56 (0%)	AV1-AV2	197	357	0.16	0.18	0.42	0.45
*East African cassava mosaic Malawi virus (B)*	13 (0%)	AC1-AC2	92	1080	0.07	0.00	0.16	0.00
	9 (0%)	AC2-AC3	260	408	0.00	0.00	-	-
	12 (0%)	AV1-AV2	191	351	0.06	0.08	0.34	0.39
*East African cassava mosaic virus (B)*	153 (3%)	AC1-AC2	92	1080	1.55	0.62	0.43	0.25
	166 (0%)	AC2-AC3	260	405	1.79	0.62	0.43	0.35
	136 (0%)	AV1-AV2	197	357	0.75	0.32	0.36	0.22
*East African cassava mosaic Zanzibar virus (B)*	14 (29%)	AC1-AC2	92	1080	0.35	0.01	0.32	0.17
	15 (0%)	AC2-AC3	260	408	0.23	0.10	0.77	0.28
	13 (0%)	AV1-AV2	197	357	0.15	0.07	0.65	0.47
*Okra enation leaf curl virus (M)*	60 (92%)	AC1-AC2	104	1089	2.96	4.66	0.05	0.25
	67 (33%)	AC2-AC3	308	453	3.45	0.68	0.32	0.04
	45 (53%)	AV1-AV2	188	348	0.38	0.75	0.21	0.50
*Pepper golden mosaic virus (B)*	54 (57%)	AC1-AC2	59	1050	1.47	0.68	0.25	0.01
	54 (0%)	AC2-AC3	254	390	1.49	0.92	0.44	0.29
*Pepper huasteco yellow vein virus (B)*	44 (2%)	AC1-AC2	80	1050	0.81	0.12	0.26	0.11
	19 (0%)	AC2-AC3	254	417	1.01	0.21	1.40	1.03
*South African cassava mosaic virus (B)*	125 (0%)	AC1-AC2	92	1080	0.55	0.60	1.04	1.47
	131 (0%)	AC2-AC3	260	408	0.56	0.42	0.70	0.43
	128 (4%)	AV1-AV2	191	351	1.16	0.99	0.24	0.08
*Sweet potato leaf curl virus (M)*	17 (41%)	AC1-AC2	92	1095	0.79	1.61	0.57	1.50
	18 (6%)	AC2-AC3	278	452	0.54	1.21	0.70	1.16
	18 (9%)	AV1-AV2	176	345	0.62	0.16	0.18	0.06
*Tomato leaf curl New Delhi virus (B)*	97 (3%)	AC1-AC2	98	1086	3.75	1.86	0.16	0.13
	88 (1%)	AC2-AC3	281	420	2.83	0.88	0.12	0.84
	105 (0%)	AV1-AV2	179	339	2.78	1.58	0.71	0.52
*Tomato yellow leaf curl virus (M)*	397 (8%)	AC1-AC2	92	1074	2.43	2.23	0.40	1.52
	588 (9%)	AC2-AC3	260	408	7.67	4.27	0.20	0.87
	623 (9%)	AV1-AV2	191	351	3.01	2.27	0.40	0.18

^1^ Number of genes with significant differences in tree length between OV and NOV regions over total number. Deviation from randomness was tested by Fisher’s exact test (*p* < 0.05). M: Monopartite; B: Bipartite. Asterisks indicate sequences formerly classified as *Bendhi yellow vein India virus*; ^2^ Number of sequences used. Percentage of recombinant sequences is shown in parentheses; ^3^ Name of the genes involved in the overlapping instance, and length of the OV region; ^4^ Length of the largest gene (internal overlapping) of the gene with 5′-terminal overlapping (5′-terminal) and of the gene with 3′-terminal overlapping (3′-terminal); ^5^ Tree length of inferred phylogenies for OV and NOV regions of each overlapping instance.
